# Modulation of expression of Connexins 37, 40 and 43 in endothelial cells in culture

**DOI:** 10.3389/fnetp.2024.1199198

**Published:** 2024-03-15

**Authors:** Wenqing Zhuang, Nick G. A. Mitrou, Steve Kulak, William A. Cupples, Branko Braam

**Affiliations:** ^1^ Division of Nephrology, Department of Medicine, Faculty of Medicine and Dentistry, University of Alberta, Edmonton, AB, Canada; ^2^ Simon Fraser University, Burnaby, BC, Canada; ^3^ Department of Physiology, Faculty of Medicine and Dentistry, University of Alberta, Edmonton, AB, Canada

**Keywords:** connexin, endothelial cell, interferon gamma, cardiovascular risk factors, JAK/STAT

## Abstract

Connexin (Cx) 37, 40, and 43 are implicated in vascular function, specifically in the electrical coupling of endothelial cells and vascular smooth-muscle cells. In the present study, we investigated whether factors implicated in vascular dysfunction can modulate the gene expression of Cx37, Cx40, and Cx43 and whether this is associated with changes in endothelial layer barrier function in human microvascular endothelial cells (HMEC-1). First, HMEC-1 were subjected to stimuli for 4 and 8 h. We tested their responses to DETA-NONOate, H_2_O_2_, high glucose, and angiotensin II, none of which relevantly affected the transcription of the connexin genes. Next, we tested inflammatory factors IL-6, interferon gamma (IFNγ), and TNFα. IFNγ (10 ng/mL) consistently induced Cx40 expression at 4 and 8 h to 10–20-fold when corrected for the control. TNFα and IL-6 resulted in small but significant depressions of Cx37 expression at 4 h. Two JAK inhibitors, epigallocatechin-3-gallate (EGCG) (100–250 μM) and AG490 (100–250 μM), dose-dependently inhibited the induction of Cx40 expression by IFNγ. Subsequently, HMEC-1 were subjected to 10 ng/mL IFNγ for 60 h, and intercellular and transcellular impedance was monitored by electric cell-substrate impedance sensing (ECIS). In response to IFNγ, junctional-barrier impedance increased more than cellular-barrier impedance; this was prevented by AG490 (5 μM). In conclusion, IFNγ can strongly induce Cx40 expression and modify the barrier properties of the endothelial cell membrane through the JAK/STAT pathway. Moreover, the Cx37, Cx40, and Cx43 expression in endothelial cells is stable and, apart from IFNγ, not affected by a number of factors implicated in endothelial dysfunction and vascular diseases.

## Introduction

Electrical information originating from the depolarization of vascular structures, including endothelial cells (ECs) and vascular smooth-muscle cells (VSMCs), propagates via gap junctions along ECs (Figueroa et al., 2003). Our interest in the physiological regulation of endothelial cell connexins originates in our studies about renal blood flow (RBF) autoregulation and the intrarenal distribution of RBF (Cupples and Braam, 2007; Hanner et al., 2010; Zehra et al., 2021). Our studies indicate that the disruption of endothelial gap junctions interferes with RBF autoregulation (Figueroa et al., 2003; Mitrou et al., 2016). Studies by others indicate that in Cx40^−/−^ mice, vascular conducted responses are impaired in all beds tested, including skeletal muscle (de Wit et al., 2000), renal microcirculation (Moller et al., 2020), and the brain (Zechariah et al., 2020), as extensively discussed by Pogoda et al. (2019). In addition, gap junctions are implicated in endothelial barrier function (Tyml, 2011). Together, Cx40 seems to play a role in microvascular regulation and barrier function. The aim of this study is to investigate the regulation of connexin expression, which forms endothelial gap junctions, particularly of Cx40, in endothelial cells.

Connexins are named by their specific protein molecular weight in kilodaltons. This report considers the vascular connexins Cx37, Cx40, and Cx43 (Larson et al., 1990; van Rijen et al., 1998). Cx40 and Cx37 are expressed in all vascular endothelia, and Cx43 is expressed in the endothelium of larger arteries and in arterial bifurcations (Saez et al., 2003; Sandow et al., 2003; Morel and Kwak, 2013; Morel, 2014). Cx43 is mainly expressed in VSMCs (Saez et al., 2003). Sorensen and Cupples pointed out that renal vascular Cx43 is variable, more often seen in cultured cells than in intact tissues (Sorensen and Cupples, 2019). There are some studies in the literature which indicate that inflammatory factors can modulate the expression of vascular connexins; yet, information on how other factors implicated in vascular disease modulate connexin expression is limited.

The hypothesis of the present study was that factors that promote cardiovascular disease, or more specifically EC dysfunction, impair the expression of EC connexins and eventually impair EC barrier function. The aim of this study was to study the responses of connexin gene expression to glucose, ANG II, oxidative stress, NO, and pro-inflammatory factors, TNFα, interferon gamma (IFNγ), and IL-6. IFNγ was identified as a factor that strongly increases Cx40 expression, and we studied whether this was mediated via the JAK/STAT pathway and whether it affected endothelial cellular barrier function.

## Methods

### Reagents and chemicals

ANG II, IL-6, TNFα, IFNγ, hydrocortisone, and the JAK/STAT inhibitors AG490 and epigallocatechin-3-gallate (EGCG) were purchased from MilliporeSigma (Darmstadt, Germany). The cell culture supplements the fetal bovine serum (FBS) and penicillin–streptomycin (10,000 U/mL). L-glutamine and medium MCDB 131 were acquired from Thermo Fisher Scientific (NY, United States). The human epidermal growth factor was purchased from VWR (IL, United States). Primers for Cx37 (Hs00704917_s1), Cx40 (Hs00979198_m1), Cx43 (Hs00748445_s1), hypoxanthine-guanine phosphoribosyltransferase (HPRT) (Hs99999909_m1), and the random primer, SuperScript^®^ II Reverse Transcriptase, TaqMan Gene Expression master mix, were obtained from Thermo Fisher Scientific (CA, United States). Details about the primary and secondary antibodies are provided in Table 1.

**TABLE 1:** Technical details about the antibodies used in the study.

	Cx37			Cx40		Cx43		
	Primary Ab	Secondary Ab		Primary Ab	Secondary Ab	Primary Ab	Secondary Ab	
Catalog #	Ab101928	Ab98488		SC-20466	A11056	MAB3068/C8093	A10056	
Lot #	GR30469-5	GR28808-1		G0711	2,090,658	NG1843011	1,292,407	
Company	Abcam	Abcam		Santa Cruz Biotechnology	Thermo Fisher	MilliporeSigma	Thermo Fisher	
Host species	Rabbit	Donkey		Goat	Donkey	Mouse	Donkey	
Target species	Human	Rabbit		Human	Goat	Rat, mouse, and human	Mouse	
Conjugate		DyLight^®^ 488			Alexa Fluor™ 546		Alexa Fluor™ 546	
Clone	Polyclonal			Polyclonal		Monoclonal		
Epitope	Second cytoplasmic domain of connexin 37/GJA4			C-terminus of connexin 40		Connexin 43 peptide (362–381)		
Stock	1:1 glycerol at −20°C	1:1 glycerol		200 μg/mL	2 mg/mL at 4°C in the dark	1:1 glycerol	2 mg/mL at 4°C in the dark	
		−20°C in the dark		−20°C		−20°C		
Dilution	1:250	1:1,000		1:100	1:500	1:250	1:500	
Control	No knockout or siRNA control			Rat Cx40 knockout (More et al., 2023)		siRNA-treated cells		
Citation	Choudhary et al. (2015)	Wang et al. (2012)		Grikscheit et al. (2008)	Rauch et al. (2018)	Hong et al. (2015)	Rauch et al. (2018)	

### Cell culture and stimulation

The immortalized human microvascular endothelial cell line (HMEC-1) was obtained from the Centers for Disease Control and Prevention (Atlanta, GA, United States). This cell line was cultured in MCDB 131 with 10% FBS, 10 ng/mL EGF, 2.5 ug/mL hydrocortisone, 1% L-glutamine, and 1% penicillin/streptomycin (10,000 U/mL) in an incubator at 37C with 5% CO_2_. The cells were routinely treated at a split ratio of 1:4 twice per week and used in passages 20–24. For the stimulation experiments, HMEC-1 cells were seeded in 35-mm tissue culture dishes at 8 × 10^5^/per well. After 24 h, the 85%–90% confluent cells were treated in a starvation medium (MCDB 131 medium with 0.5% FBS and 1% penicillin/streptomycin, 10,000 U/mL) overnight. The 95%–100% confluent cells were used for the stimulation experiments during the next day.

Additional experiments were performed in HUVECs, passages 3–5, and glomerular cultured ECs (GCECs), passages 3–7, to study the response to IFNγ. HUVECs were kindly provided by Dr. Allan Murray (University of Alberta) and incubated in the M199 medium with 20% FBS, 1% penicillin/streptomycin, 1% L-glutamine, and 10 mg/mL (BD 356006) ECGS at 37°C and 5% CO_2_. Cell passage 2–5 cells were used for the stimulation experiment. GCECs were purchased from Angio-Proteomie (Boston, MA) and were cultured in EGM™-2 MV BulletKit media (Lonza, Walkersville, MD) in a 37°C, 5% CO_2_ incubator. Cell passage 3–7 cells were used for the experiments.

The various compounds, glucose, 5 mM and 25 mM; DETA-NONOate, 1 μM and 10 μM (Braam et al., 2004); ANG II, 10 nM and 100 nM (Braam et al., 2003); IL-6, 20 U/mL (Bluyssen et al., 2010a); TNFα, 20 ng/mL (Hao et al., 2005); and IFNγ 1, 10 and 30 ng/mL (Bluyssen et al., 2010a), were added to the desired wells. Phosphate-buffered saline (D-PBS) was used as a control. The cells were cultured at 37°C in an incubator with 5% CO_2_ for 4–8 h. In the experiments where the JAK/STAT blockers, AG490 and EGCG, were used, the compounds were added 1 h before stimulation with IFNγ.

### HMEC-1 immunofluorescence

Twelve-well plates were coated with fibronectin, 0.5 ug/mL, for 1 h; then, 3 × 10^3^ HMEC-1 cells were seeded into every well and incubated overnight to reach 80% confluency. The cells were fixed with 4% paraformaldehyde in PBS for 10 min at room temperature, followed by a wash in calcium free D-PBS with 0.2% Triton X-100. The cultures were blocked in 10% donkey serum and 1% BSA in TPBS for 1 hour and then blotted in primary connexin antibodies at 4°C overnight. Incubation with the secondary antibodies was carried out with donkey anti-rabbit IgG DyLight 488 (1:1,000 dilution) for the primary antibodies to Cx37, donkey anti-goat IgG Alexa Fluor™ 546 (1:500 dilution) for the primary antibodies to Cx40, and donkey anti-mouse IgG Alexa Fluor™ 546 (1:500 dilution) for the primary antibodies to Cx43, all for 1 hour at room temperature. The cells were stained with 0.1 ug/mL DAPI for 3 min, then mounted on the slide, and stocked in 4°C, ready for examination by fluorescence microscopy. The stained slides were imaged with the Quorum WaveFX spinning-disc confocal system (Quorum Technologies) using Volocity 6.3.1 software.

### RNA extraction and real-time PCR

Total RNA was extracted using the RNeasy Mini Kit (QIAGEN, MD, United States), following standard procedures. The total RNA quantity and quality were assessed using a NanoDrop 2000 spectrophotometer (Thermo Scientific, DE, United States). Reverse transcription was carried out using SuperScript^®^ II Reverse Transcriptase with random primers. The amount of total RNA was 1 ug/per RT reaction.

PCR was performed using a 7500 Real-Time PCR System (Thermo Fisher Scientific, CA, United States) with TaqMan Cx37, Cx40, and Cx43 primers and the TaqMan Gene Expression master mix. PCR was performed as follows: an initial denaturing step was carried out at 95°C for 10 min, along with 40 cycles at 95°C for 15 s and 60°C for 1 m. The HPRT expression was used for normalization, and fold change was calculated using ΔΔCT.

### ECIS

The endothelial barrier function was assessed in real time, recording cellular- and junctional-barrier electric impedance using the Z-Theta ECIS system (Applied Biophysics, NY, United States). A gold 8W10E PET array (Applied Biophysics, NY, United States) was treated with 10 mM L-cysteine for 0.5 h. A 400 ul, 1.1 × 10^5^ cells/mL HMEC-1 suspension was seeded into each well on the array; one well was left cell-free with only medium. The cells were incubated for 4 h at 37°C and 5% CO_2_ to attach and reach 80%–90% confluence. The growth medium was removed, and the cells were washed with a calcium-free, phosphate-buffered saline and left in the 0.5% FBS starvation medium overnight. After the array was placed on the stage to connect the recording system, an equilibration period was observed for 1 h. Then, the medium was replaced by the growth medium with or without stimulants in each desired well and recording was started. The impedance of the cell-electrode cartridge was measured through the ECIS Z-Theta system using the multiple-frequency option (the frequency range was from 25 Hz to 64 KHz). Impedance was continuously recorded for 60 h using ECIS software (1.2.123). Impedance time series were normalized to the fifth hour of recording. The 4 kHz and 64 kHz normalized impedance data were exported for statistical analysis.

### Data analysis

Gene expression data are shown as the mean ± SD. Differences between the means were analyzed using Prism 8 software by a two-way analysis of variance (ANOVA) followed by multiple comparisons by the Tukey–Kramer test (GraphPad, San Diego, CA). For the analysis of the ECIS data, the baseline was calculated as the average of the values measured in the fifth hour, and the difference between this baseline and all remaining data points up to 65 h was calculated. These values corrected for the baseline were subjected to nonlinear regression analysis using a four-parameter sigmoidal fit. The parameters were used to recalculate the fitted curves for the 5–60 h and represented as the mean ±95% confidence interval. The level, where ECIS reached a plateau for the four and kHz frequencies, was calculated as the average of the hourly impedance data from the time point of 40–50 h for each observation. This plateau was compared using one-way ANOVA followed by the Tukey–Kramer test. The threshold for statistical significance was set at *p* <0.05.

## Results

### Connexin gene expression in endothelial cells in a culture is not dependent on the confluency

**FIGURE 1:**
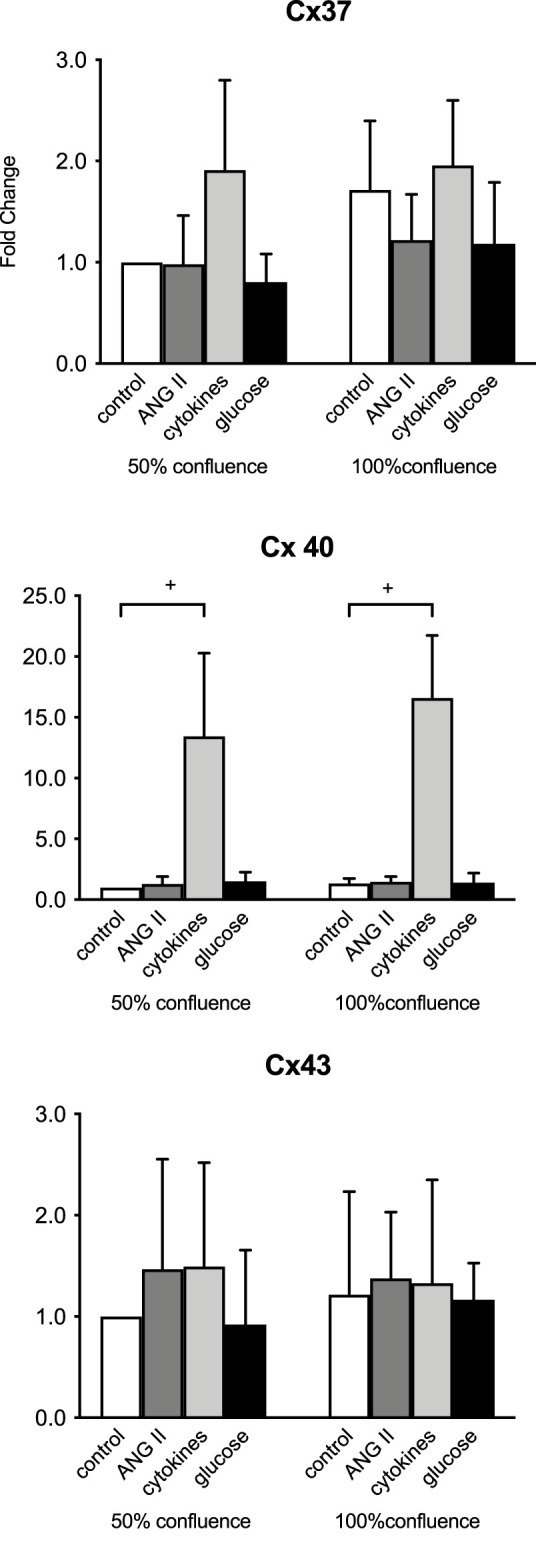
Initial evaluation of connexin mRNA responses at 50 versus 100% confluency in time-control cells and in ECs after 8 h of exposure to 100 nM angiotensin II (ANG II; dark gray), a combination of 20 IU/mL IL-6, 10 ng/mL IFNγ, and 20 ng/mL TNFα (cytokines, light gray) and 20 mmol/L glucose (glucose, black). +: *p* <0.0001. Data represent the mean ± SD of three independent experiments.

In the initial studies, we assessed the gene and protein expression of Cx37, Cx40, and Cx43 in HMEC-1 cells. Figure 1 compares the gene expression in HMEC-1 at 50% and 100% confluency. Under unstimulated conditions, the gene expression of the three connexins was not different between the cells at 50% or 100% confluency (Figure 1), nor did the confluency influence responses to ANG II, high glucose, or combined IL-6, IFNγ, and TNFα. The strong (note the different scales) induction of Cx40 upon cytokines was unchanged (Figure 1). Subsequent experiments were performed at a confluence of 90%–100%.

### Connexin proteins are expressed in endothelial cells in a culture

**FIGURE 2:**
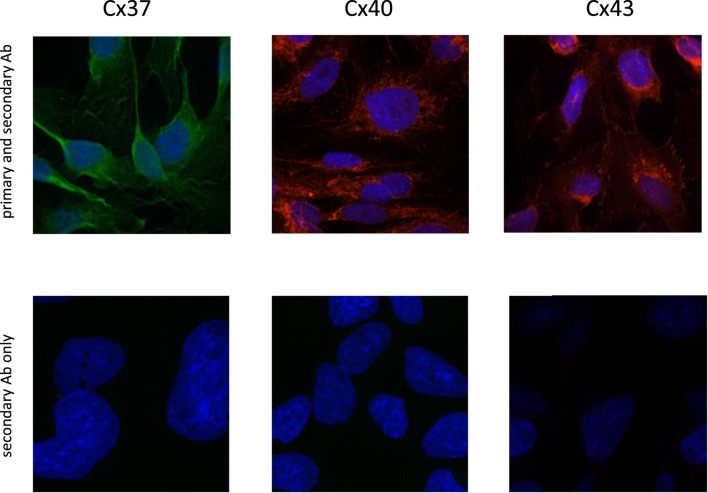
Immunofluorescence of connexin 37 (Alexa Fluor™ 488, green), 40 (Alexa Fluor™ 594, red), and 43 (Alexa Fluor™ 594, red) which were stained in human microvascular ECs (HMEC-1; X-200) (upper panels). Cell nuclei were stained with DAPI (dark blue fluorescence). Confocal images of cells exposed only to the secondary antibody did not reveal any signal for Cx37, Cx40, and Cx43 (lower panels).

Confocal imaging (Figure 2) showed that the green positive Cx37 signal was distributed throughout the cells; this was unexpected and might point toward a protein trafficking issue in our cell line for Cx37. The red punctate signal of Cx40 and Cx43 immunofluorescence on HMEC-1 appeared at the cell surface. The slides treated only with the secondary Abs were negative.

### HMEC-1 Cx37, Cx40, and Cx43 gene expression responses to ANG II, DETA-NONOate, glucose, and H_2_O_2_

**FIGURE 3:**
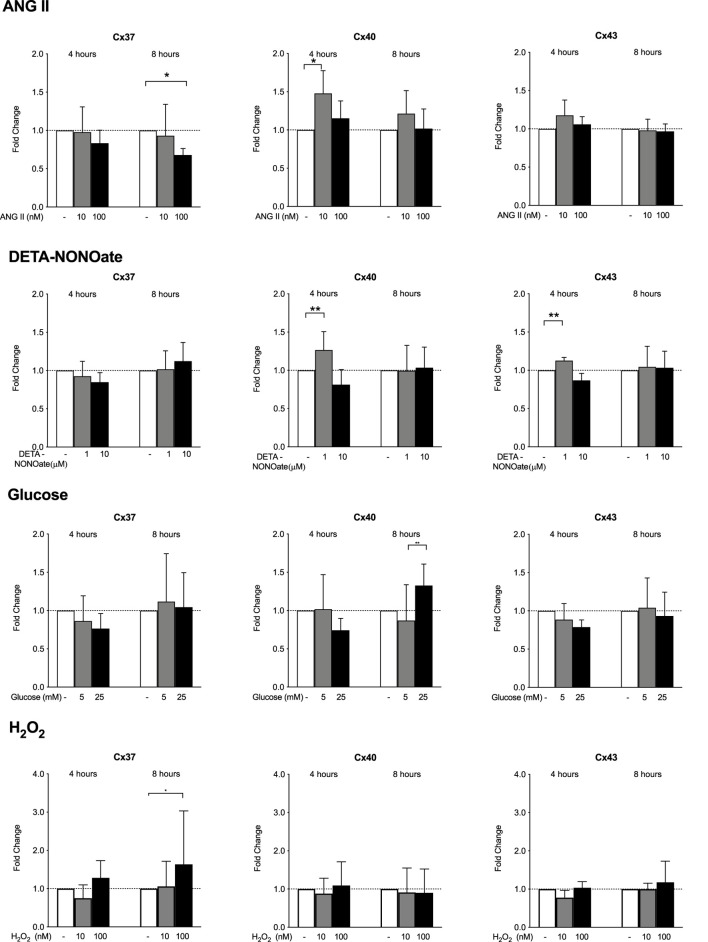
HMEC-1 mRNA expression of Cx37, Cx40, and Cx43 upon exposure to ANG II, DETA-NONOate, glucose, and H_2_O_2_. Cx37 gene expression decreased after 8 h of exposure to 100 μM ANG II. Cx40 expression mildly increased after 4 h of exposure to 10 μM ANG II. The expression of Cx43 remained stable. Concentrations of 1 µM and 10 µM DETA-NONOate did not affect Cx37 expression. DETA-NONOate at 1 μM increased the Cx40 and Cx43 gene expression after 4 h. Cx37, Cx40, and Cx43 gene expressions did not change upon exposure to 5 or 25 mM glucose compared to the control. In response to H_2_O_2_, there was a slight induction of Cx37 after 8 h of exposure to 100 nM. It should be noted that all observed changes were quantitatively small. All data were derived from six independent experiments per experimental setting and three replicates in each experiment. *p <0.05; **p <0.01.

Exposure to the high dose of 100 uM ANG II for 8 h decreased the Cx37 gene expression. Exposure of HMEC-1 to 10 μM ANG II significantly increased Cx40 expression after 4 h but not after 8 h of stimulation (Figure 3). ANG II did not affect the Cx43 gene expression at 4 or 8 h. DETA-NONOate did not affect the Cx37 expression. A volume of 1 μM DETA-NONOate and not 10 μM elevated the Cx40 and Cx43 expression after 4 h of exposure only. Stimulating HMEC-1 with 5 and 25 mM glucose did not result in the modulation of the Cx37, Cx40, and Cx43 gene expression. The 8-hour exposure of the cells to 100 nM H_2_O_2_ led to a small but significant increase in the Cx37 expression. All-in-all, the changes upon stimulation were absent or very small in all these compounds.

### HMEC-1 Cx37, Cx40, and Cx43 gene expression responses to pro-inflammatory cytokines

**FIGURE 4:**
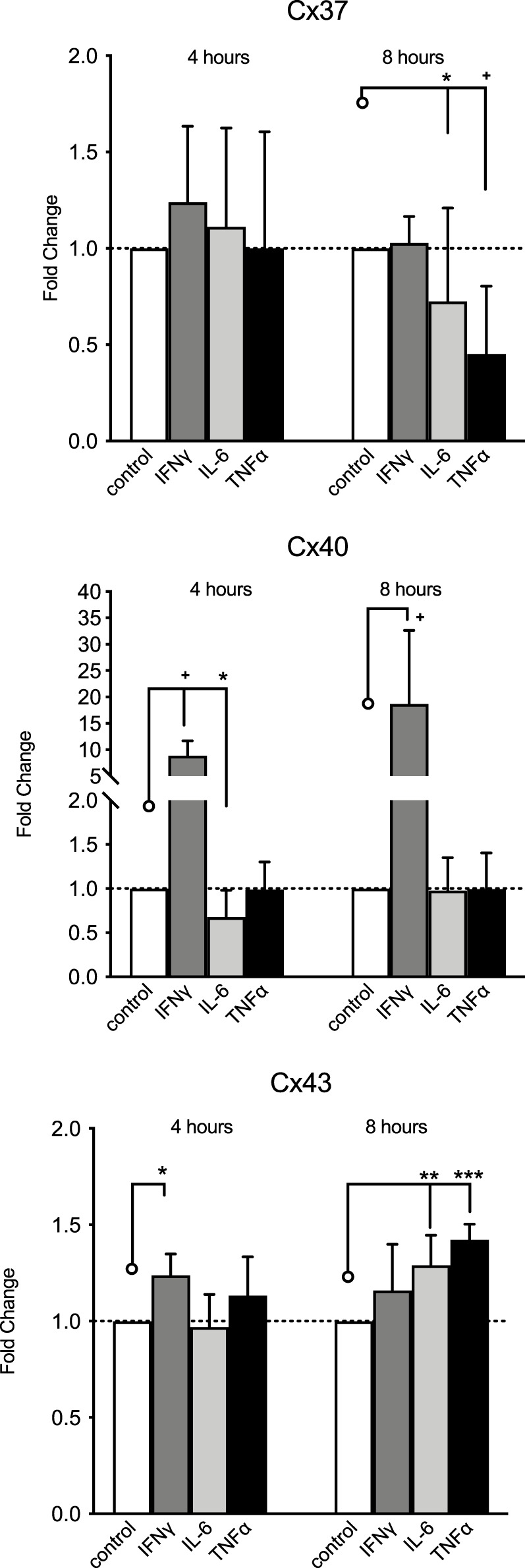
HMEC-1 mRNA expression of Cx37, Cx40, and Cx43 upon exposure to TNFα, IFNγ, and IL-6. Cx37 expression significantly decreased after 8 h of exposure to IL-6 (20 U/mL) and TNFα (20 ng/mL) modulation. Cx40 expression was reduced after 4 h of exposure to IL-6, but not after 8 h. IFNγ (10 ng/mL) induced Cx40 expression, approximately 10-fold after 4 h and approximately 17-fold at 8 h. IFNγ increased Cx43 expression at 4 h but not at 8 h. Both TNFα (*p* <0.001) and IL-6 increased Cx43 expression significantly after 8 h of stimulation. All data were derived from six independent experiments per experimental setting and three replicates in each experiment. *p <0.05, **p <0.01, ***p <0.001, and + *p* < 0.0001. Significance is indicated as compared to the bars pointed at with a line with a circle.

Both IL-6 (20 U/mL) and TNFα (20 ng/mL) decreased HMEC-1 Cx37 gene expression and increased Cx43 expression but only after 8 h of exposure (Figure 4). IL-6 reduced Cx40 expression after 4 h, but not after 8 h of exposure. While IFNγ did not modulate Cx37 expression, 10 ng/mL IFNγ expression increased Cx40 nearly 10-fold after 4 h and 17-fold after 8 h of exposure. IFNγ 10 ng/mL minimally increased HMEC-1 Cx43 gene expression (by 20%) after 4 h of exposure.

### IFNγ induces Cx40 expression via the JAK/STAT pathway

**FIGURE 5:**
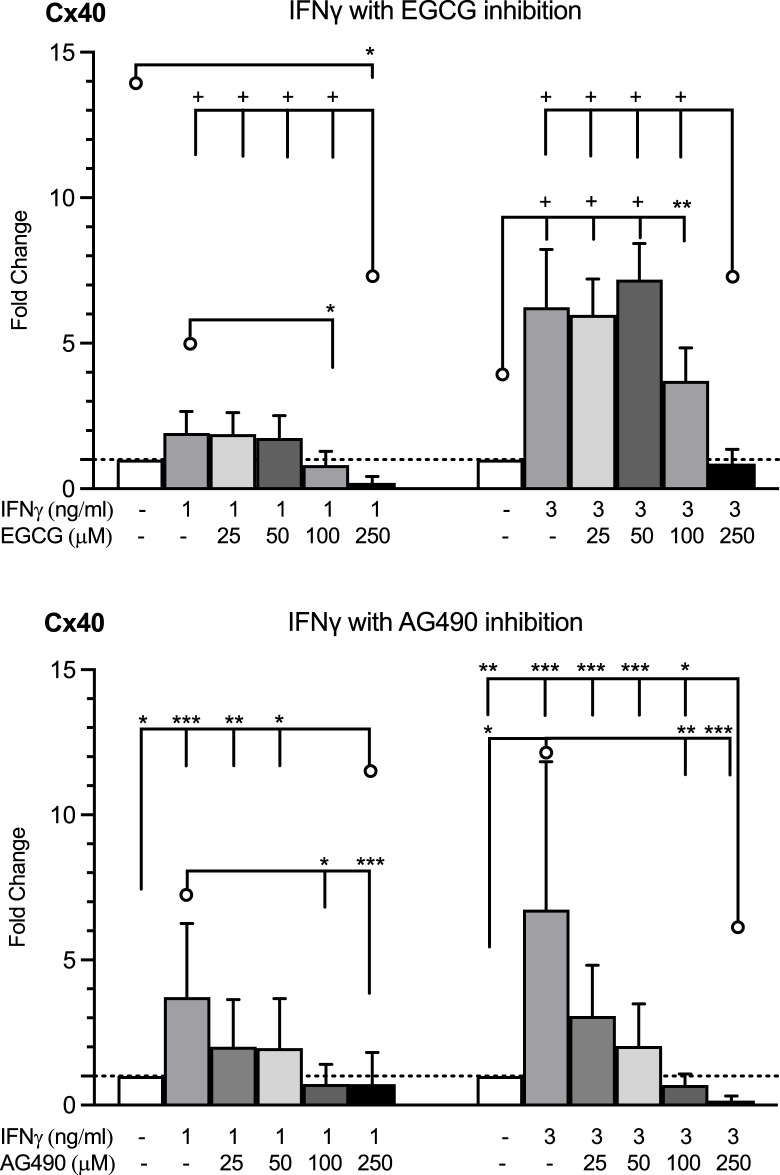
HMEC-1 Cx40 mRNA expression in response to IFNγ in the absence and presence of JAK/STAT inhibitors, EGCG and AG490. EGCG inhibited the enhanced Cx40 gene expression by IFNγ 1 or 3 ng/mL at concentrations over 100 μM. Every concentration of AG490 inhibited the elevated Cx40 expression induced by IFNγ in a dose of 1 or 3 ng/mL. The highest dosages of the EGCG and AG490 inhibition resulted in a decreased Cx40 gene expression versus control. All data were derived from three independent experiments per experimental setting and three replicates in each experiment. A total of 95% confidence intervals were derived from the curve fitting. *p <0.05, **p <0.01, ***p <0.001, and + *p* < 0.0001. Significance is indicated as compared to the bars pointed at with a line with a circle.

Since IFNγ signals are propagated via the JAK/STAT pathway, we tested whether the observed induction of Cx40 by 1 or 3 ng/mL of IFNγ could be blunted by EGCG and AG490, inhibitors of the JAK/STAT pathway. Pre-treatment with each compound for 1 h prior to stimulation with IFNγ prevented the IFNγ-induced induction of Cx40 expression, albeit only at a higher concentration of EGCG. HMEC-1 co-cultured with the AG490 concentration exceeding 100 μM or EGCG concentration of 250 μM displayed decreased Cx40 gene expression compared to the control (Figure 5). This would imply that there is, to a certain extent, a baseline JAK/STAT-dependent Cx40 gene expression, not necessarily driven by IFNγ.

### Stimulation of Cx40 by IFNγ in HUVECs and GCECs

To demonstrate that the stimulation of Cx40 expression was not unique for the HMEC-1 cell line, we performed limited experiments in HUVECs (*n* = 3) and GCECs (*n* = 3). In HUVECs, we observed a limited 2.0 ± 0.3-fold induction of Cx40 gene expression to 10 ng/mL IFNγ after 4 h. In GCECs, we observed a stronger induction after exposure for 4 h to 30 ng/mL of 27 ± 1-fold. These experiments show that the induction of Cx40 by IFNγ is not unique to HMEC-1.

### IFN γ dose-dependently affects EC barrier function

**FIGURE 6:**
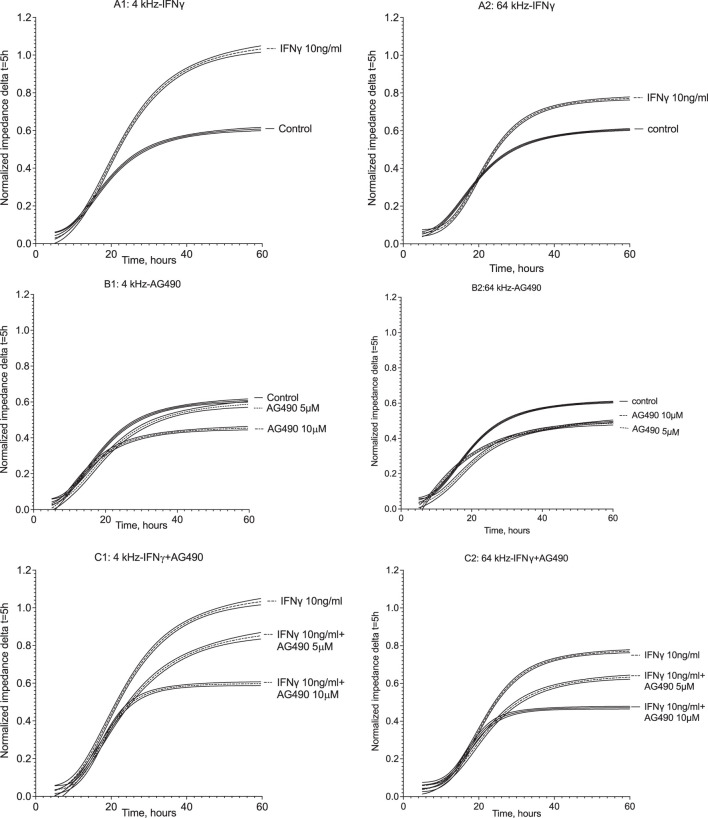
Normalized impedance of HMEC-1 at 4 kHz and 64 kHz throughout 60 h of treatment. Panels A1 and A2 show impedance changes in response to IFNγ at 4 and 64 kHz, respectively. After 1 h of recording, the medium was replaced with the growth medium containing 10 ng/mL IFNγ with DPBS serving as the control. IFNγ resulted in a higher impedance level compared to the control after 30 h at both frequencies. Panels B1 and B2 show impedance time series at 4 kHz and 64 kHz, respectively, where the HMEC-1 medium was replaced with the growth medium containing AG490 5 μM and 10 uM, with DPBS as the control. At 4 kHz, impedance plateaued at similar levels to the control in the presence of 5 μM AG490; yet, the plateau in the presence of 10 uM AG490 was lower compared to the control. At 64 kHz, both doses of AG490 resulted in a lower impedance plateau compared to the control. In panels C1 and C2, the response at 4 kHz and 64 kHz is shown where HMEC-1 cells were pretreated with AG490 and then exposed to 10 ng/mL IFNγ. Both 5 μM and 10 μM AG490 diminished the enhanced impedance in response to 10 ng/mL IFNγ at both 4 kHz and 64 kHz. Data were derived from 5–12 wells in the ECIS arrays in 5–7 independent experiments.

**FIGURE 7:**
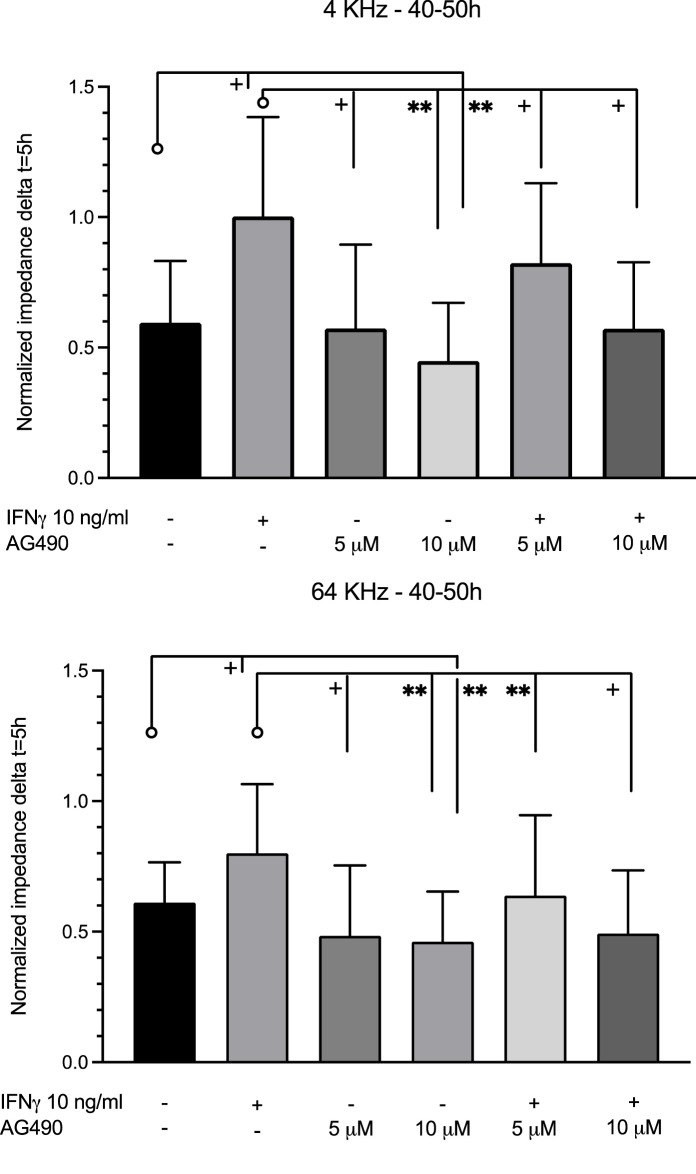
Comparison of the average impedance level between 40 and 50 h at 4 and 64 kHz. At 4 kHz between 40 and 50 h, IFNγ significantly increased impedance compared to the control. Pre-treatment with 10 μM AG490 decreased the impedance level below the control level. Pre-treatment with both doses of AG490 diminished the enhancement of impedance by IFNγ. At 64 kHz between 40 and 50 h, changes were similar but less pronounced. Data were obtained from 5–7 independent experiments. **: *p* <0.005; +: *p* <0.0001. Significance is indicated as compared to the bars pointed at with a line with a circle.

The multiple-frequency ECIS system enables the recording of real-time changes in HMEC-1 impedance during IFNγ exposure. During the 60 h of impedance recording at 4 kHz, which assesses trans-junctional impedance, IFNγ strongly increased cell impedance (Figure 6A and Figure 7). In the first 30 h, impedance in HMEC-1 exposed to IFNγ was two times higher than that in untreated HMEC-1. After the cells became confluent, impedance plateaued. As shown in Figure 6A and Figure 7, IFNγ also caused significant changes in impedance when studied at the higher, 64 kHz, frequency, which assess transcellular impedance, but the effects were less pronounced (Figure 7). Therefore, the clear increased impedance at the lower frequency by IFNγ stimulation likely results from changes in the composition of the intercellular junction.

Incubation of HMEC-1 with the JAK/STAT inhibitor AG490 at 5 µM did not affect real-time impedance versus the untreated cells at both 4 kHz and 64 kHz (Figure 6B; Figure 7). At 10 µM, however, impedance was lower than that in control cells at both frequencies. The cells pre-exposed to AG490 at 5 or 10 µM followed by IFNγ stimulation displayed the dose-dependent attenuation of the increase in impedance elicited by IFNγ at 4 kHz and 64 kHz (Figure 6C; Figure 7).

## Discussion

Connexins are the building blocks of gap junctions (connexins) that enable intercellular, and particularly axial, electrical communication in endothelia. Previous studies implicating an important role for endothelial Cx40 in the autoregulation of the RBF (Oppermann et al., 2013; Mitrou et al., 2016; Zehra et al., 2021; More et al., 2023) triggered the current investigation. This study tested whether factors implicated in endothelial cell dysfunction and in cardiovascular and kidney disease affect the expression of vascular connexins, particularly of Cx40. ANG II mildly stimulated Cx40 expression, but not other connexins, and this was not sustained. The NO donor, DETA-NONOate, minimally stimulated the Cx40 and Cx43 expression, but this was not sustained in time either. The high glucose concentration minimally stimulated Cx40.

More importantly, IFNγ strongly enhanced the endothelial Cx40 expression, and this is mediated via the JAK/STAT pathway. In addition, TNFα stimulated Cx43 expression, but this was minimal. The effects of IFNγ on Cx40 may translate into an increase in EC barrier function, as assessed by measurements of electrical impedance. Intercellular more than transcellular impedance increased upon IFNγ administration, and this was also dependent on the JAK/STAT pathway. All in all, the most important finding is the stability of the vascular connexin expression, showing little or no modulation by several factors implicated in the microcirculatory function (ANG II and DETA–NONOate) and dysfunction (glucose, H_2_O_2_, and cytokines). The only exception was the strong effect of IFNγ on Cx40 expression.

As mentioned, our study was triggered by observations about connexins in the kidney. Endothelial connexins in the kidney serve several functions, with Cx40 and, possibly, Cx37 being mainly responsible for vascular conducted responses, elicited by renal autoregulation (Hanner et al., 2010; Zehra et al., 2021). Outside of the kidney, Cx40 is widespread in the vasculature, and the knockout of Cx40 is associated with the reduced spread of calcium signals, irregular vasomotion, and importantly, electrically mediated vascular-conducted responses (Zechariah et al., 2020) [for an extensive review, see the work of Pohl (2020)]. Cx40 is an important component of the regulation of renin release (Takenaka et al., 2008). Cx40 and Cx37 can have anti-inflammatory actions in the endothelium, while Cx43 has been implicated in pro-inflammatory actions (Abed et al., 2014). The modulation of the expression of these connexins has been less studied. Of the factors implicated in endothelial dysfunction and microvascular regulation, the most pronounced findings were that 1) Cx40 is induced by IFNγ, 2) the induction of Cx40 by IFNγ is mediated via the JAK/STAT pathway, and 3) this possibly affects the EC barrier function. We have been unable to identify any studies in the literature about these observations.

Teleologically, it makes sense that IFNγ activates the anti-inflammatory connexin Cx40. In a rat model of sepsis induced by cecal ligation and perforation, the enhanced expression of Cx40 was observed in the aortic endothelium during the recovery phase (Rignault et al., 2005). In rats injected with a single dose of LPS, aortic Cx40 expression was also induced. The administration of omega-3 fatty acids prevented the increase in Cx40 (Frimmel et al., 2014). In a follow-up study, the same group showed that LPS injected into hereditary hyper-triglyceridemic rats decreased aorta Cx40. Omega-3 fatty acids, in this case, were associated with increases in Cx40 (Frimmel et al., 2016). Specific to vascular-conducted responses, LPS administration reduced electrical coupling between endothelial cells in the vasculature of WT but not Cx40^−/−^ mice (Siddiqui et al., 2015). Taken together, inflammation induced by LPS induced Cx40 expression, but seems to be dependent on other conditions. Unfortunately, a specific role for IFNγ was not investigated in these studies.

JAK/STAT signaling was implicated in studies in the corneal EC (Hara et al., 2019). IFNγ increased phosphorylated STAT3, consistent with the results from our group in the microvascular EC in a culture (Bluyssen et al., 2010b). Trans-endothelial resistance dose-dependently decreased when anti-STAT3 antibodies were applied, indicating a protective role for STAT3 in maintaining the EC barrier function. This was also associated with a decrease in the tight junction molecule zona occludens-1 (ZO-1). These observations are in line with our current observation that IFNγ increased EC barrier impedance and the inhibition of JAK/STAT signaling abrogated this effect. The analysis of the upstream 500 bp region of the Cx40 gene using ConTra v3 (Kreft et al., 2017) revealed binding sites for STAT1. Taken together, the role of JAK/STAT signaling in the regulation of Cx40 by IFNγ is suggested by the current observations, but this would need to be further substantiated in further studies addressing Cx40 protein expression and function.

We assessed junctional and cellular endothelial barriers by electric cell-substrate impedance sensing; intercellular impedance clearly increased upon exposure to IFNγ. Again, there is plenty of information about LPS in modulating intercellular resistance, and the picture is complex regarding the role of Cx40 in the changes in impedance (Tyml, 2011). Few studies have looked at the effect of cytokines on trans-endothelial impedance (or resistance). Confusingly, one study reported opposite findings, where over a similar time frame and using similar dosages of IFNγ, trans-endothelial resistance decreased, indicating a disruption of the endothelial barrier (Ehlers et al., 2023). In another study, it was determined that endothelial impedance was decreased by the plasma from a patient infected with SARS-CoV-2, indicating a disruption of the barrier. IFNγ levels were increased in these patients, but neutralizing antibodies against IFNγ did not change the decreased impedance (Kovacs-Kasa et al., 2022). Despite these contrasting observations, the role of IFNγ to increase EC barrier function is likely from our ECIS observations and could be linked to increased Cx40 expression. The observed decrease in impedance upon JAK/STAT inhibition under unstimulated conditions indicates a role of JAK/STAT signaling in EC barrier function.

The most important finding of the current study is that the endothelial connexin expression is not affected in any significant way by variations in the levels of factors which have been implicated in endothelial dysfunction, except for IFNγ. Since vascular connexins are integral to the autoregulation of the blood flow and vascular conducted responses (Hanner et al., 2010; Abed et al., 2014), the stable expression would favor vascular function to remain intact in a wide set of circumstances. The enhanced expression of Cx40 upon exposure to IFNγ can be viewed as an anti-inflammatory response. Cx40 has been associated with anti-inflammatory properties in other settings, which would be protective for endothelial function. Taken together, the current findings indicate that the expression of vascular connexins is stable upon the exposure of endothelial cells to a number of stimuli implicated in vascular dysfunction.

There are several limitations to our studies. First, we used the HMEC-1 cell line, which has been characterized in the past (Bouis et al., 2001). Nevertheless, this cell line does not represent a particular segment of the vascular bed. We observed a similar induction of Cx40 expression by IFNγ in HUVECs and GCECs. We, therefore, consider it likely that this response to IFNγ is a generic response of EC and not necessarily related to a particular location in the vascular bed or limited to a particular EC subtype. The study indicates that these three endothelial cell types respond to IFNγ similarly. Whether this eventually translates into an altered gap junction function *in vivo* requires different studies. We studied mRNA levels; mRNA levels may, but do not necessarily, reflect the altered protein, with mRNA often being observed both with and without the corresponding changes in the protein expression. Our ECIS studies show a clear increase in trans-junctional impedance upon the exposure of HMEC-1 to IFNγ, which could be related to the increase in Cx40. However, further studies would be needed to make this conclusion firm. To fully establish that the IFNγ-induced increase in Cx transcription leads to Cx40 protein formation, the incorporation of the protein in the gap junctions, in conjunction with co-factors, requires extensive further study. Moreover, to demonstrate that the responses will sustain for a longer duration and will affect EC function for a longer duration would require significant investment using animal models.

Taken together, this exploration of the modulation of endothelial connexin expression reveals a strong induction of Cx40 by IFNγ via the JAK/STAT pathway. Other factors implicated in endothelial (dys)function did not affect Cx37, Cx40, and Cx43 expression significantly. In addition, IFNγ via JAK/STAT increases monolayer impedance, likely acting on the junctional barrier. Further studies are needed to investigate whether the strong enhancement of Cx40 transcription is associated with protein expression, with functional changes in the gap junction and, finally, with any relevance of these findings *in vivo*.

## Data Availability

The raw data supporting the conclusion of this article will be made available by the authors, without undue reservation.

## References

[B1] Abed A. Dussaule J. C. Boffa J. J. Chatziantoniou C. Chadjichristos C. E. (2014). Connexins in renal endothelial function and dysfunction. Cardiovasc Hematol. Disord. Drug Targets 14, 15–21. 10.2174/1871529x14666140401105827 24720461

[B2] Bluyssen H. A. Rastmanesh M. M. Tilburgs C. Jie K. Wesseling S. Goumans M. J. (2010a). IFN gamma-dependent SOCS3 expression inhibits IL-6-induced STAT3 phosphorylation and differentially affects IL-6 mediated transcriptional responses in endothelial cells. Am. J. Physiol. Cell Physiol. 299, C354–C362. 10.1152/ajpcell.00513.2009 20484656

[B3] Bluyssen H. A. Rastmanesh M. M. Tilburgs C. Jie K. Wesseling S. Goumans M. J. (2010b). IFN gamma-dependent SOCS3 expression inhibits IL-6-induced STAT3 phosphorylation and differentially affects IL-6 mediated transcriptional responses in endothelial cells. Am. J. Physiol. Cell Physiol. 299, C354–C362. 10.1152/ajpcell.00513.2009 20484656

[B4] Bouis D. Hospers G. A. Meijer C. Molema G. Mulder N. H. (2001). Endothelium *in vitro*: a review of human vascular endothelial cell lines for blood vessel-related research. Angiogenesis 4, 91–102. 10.1023/a:1012259529167 11806248

[B5] Braam B. Allen P. Benes E. Koomans H. A. Navar L. G. Hammond T. (2003). Human proximal tubular cell responses to angiotensin II analyzed using DNA microarray. Eur. J. Pharmacol. 464, 87–94. 10.1016/s0014-2999(03)01382-7 12620499

[B6] Braam B. De Roos R. Dijk A. Boer P. Post J. A. Kemmeren P. P. (2004). Nitric oxide donor induces temporal and dose-dependent reduction of gene expression in human endothelial cells. Am. J. Physiol. Heart Circ. Physiol. 287, H1977–H1986. 10.1152/ajpheart.00323.2004 15242832

[B7] Choudhary M. Naczki C. Chen W. Barlow K. D. Case L. D. Metheny-Barlow L. J. (2015). Tumor-induced loss of mural Connexin 43 gap junction activity promotes endothelial proliferation. BMC Cancer 15, 427. 10.1186/s12885-015-1420-9 26002762 PMC4464240

[B8] Cupples W. A. Braam B. (2007). Assessment of renal autoregulation. Am. J. Physiol. Ren. Physiol. 292, F1105–F1123. 10.1152/ajprenal.00194.2006 17229679

[B9] de Wit C. Roos F. Bolz S. S. Kirchhoff S. Kruger O. Willecke K. (2000). Impaired conduction of vasodilation along arterioles in connexin40-deficient mice. Circ. Res. 86, 649–655. 10.1161/01.res.86.6.649 10747000

[B10] Ehlers H. Nicolas A. Schavemaker F. Heijmans J. P. M. Bulst M. Trietsch S. J. (2023). Vascular inflammation on a chip: a scalable platform for trans-endothelial electrical resistance and immune cell migration. Front. Immunol. 14, 1118624. 10.3389/fimmu.2023.1118624 36761747 PMC9903066

[B11] Figueroa X. F. Paul D. L. Simon A. M. Goodenough D. A. Day K. H. Damon D. N. (2003). Central role of connexin40 in the propagation of electrically activated vasodilation in mouse cremasteric arterioles *in vivo* . Circ. Res. 92, 793–800. 10.1161/01.RES.0000065918.90271.9A 12637364

[B12] Frimmel K. Sotnikova R. Navarova J. Bernatova I. KriZak J. Haviarova Z. (2016). Omega-3 fatty acids reduce lipopolysaccharide-induced abnormalities in expression of connexin-40 in aorta of hereditary hypertriglyceridemic rats. Physiol. Res. 65 (Suppl. 1), S65–S76. 10.33549/physiolres.933401 27643941

[B13] Frimmel K. Vlkovicova J. Sotnikova R. Navarova J. Bernatova I. Okruhlicova L. (2014). The effect of omega-3 fatty acids on expression of connexin-40 in Wistar rat aorta after lipopolysaccharide administration. J. Physiol. Pharmacol. 65, 83–94. 24622833

[B14] Grikscheit K. Thomas N. Bruce A. F. Rothery S. Chan J. Severs N. J. (2008). Coexpression of connexin 45 with connexin 43 decreases gap junction size. Cell Commun. adhesion 15, 185–193. 10.1080/15419060802013943 18649189

[B15] Hanner F. Sorensen C. M. Holstein-Rathlou N. H. Peti-Peterdi J. (2010). Connexins and the kidney. Am. J. Physiol. Regul. Integr. Comp. Physiol. 298, R1143–R1155. 10.1152/ajpregu.00808.2009 20164205 PMC2867516

[B16] Hao J. L. Suzuki K. Lu Y. Hirano S. Fukuda K. Kumagai N. (2005). Inhibition of gap junction-mediated intercellular communication by TNF-alpha in cultured human corneal fibroblasts. Invest. Ophthalmol. Vis. Sci. 46, 1195–1200. 10.1167/iovs.04-0840 15790879

[B17] Hara S. Tsujikawa M. Maruyama K. Nishida K. (2019). STAT3 signaling maintains homeostasis through a barrier function and cell survival in corneal endothelial cells. Exp. eye Res. 179, 132–141. 10.1016/j.exer.2018.11.008 30439348

[B18] Hong X. Sin W. C. Harris A. L. Naus C. C. (2015). Gap junctions modulate glioma invasion by direct transfer of microRNA. Oncotarget 6, 15566–15577. 10.18632/oncotarget.3904 25978028 PMC4558171

[B19] Kovacs-Kasa A. Zaied A. A. Leanhart S. Koseoglu M. Sridhar S. Lucas R. (2022). Elevated cytokine levels in plasma of patients with SARS-CoV-2 do not contribute to pulmonary microvascular endothelial permeability. Microbiol. Spectr. 10, e0167121. 10.1128/spectrum.01671-21 35171047 PMC8849075

[B20] Kreft L. Soete A. Hulpiau P. Botzki A. Saeys Y. De Bleser P. (2017). ConTra v3: a tool to identify transcription factor binding sites across species, update 2017. Nucleic Acids Res. 45, W490–W494. 10.1093/nar/gkx376 28472390 PMC5570180

[B21] Larson D. M. Haudenschild C. C. Beyer E. C. (1990). Gap junction messenger RNA expression by vascular wall cells. Circ. Res. 66, 1074–1080. 10.1161/01.res.66.4.1074 1690612

[B22] Mitrou N. Braam B. Cupples W. A. (2016). A gap junction inhibitor, carbenoxolone, induces spatiotemporal dispersion of renal cortical perfusion and impairs autoregulation. Am. J. Physiol. Heart Circ. Physiol. 311, H582–H591. 10.1152/ajpheart.00941.2015 27371687

[B23] Moller S. Jacobsen J. C. B. Holstein-Rathlou N. H. Sorensen C. M. (2020). Lack of connexins 40 and 45 reduces local and conducted vasoconstrictor responses in the murine afferent arterioles. Front. Physiol. 11, 961. 10.3389/fphys.2020.00961 32848881 PMC7431600

[B24] More H. L. Braam B. Cupples W. A. (2023). Reduced tubuloglomerular feedback activity and absence of its synchronization in a connexin40 knockout rat. Front. Netw. Physiol. 3, 1208303. 10.3389/fnetp.2023.1208303 37705697 PMC10495682

[B25] Morel S. (2014). Multiple roles of connexins in atherosclerosis- and restenosis-induced vascular remodelling. J. Vasc. Res. 51, 149–161. 10.1159/000362122 24853725

[B26] Morel S. Kwak B. R. (2013). Vascular connexins in restenosis after balloon injury. Methods Mol. Biol. 1037, 381–398. 10.1007/978-1-62703-505-7_22 24029948

[B27] Oppermann M. Carota I. Schiessl I. Eisner C. Castrop H. Schnermann J. (2013). Direct assessment of tubuloglomerular feedback responsiveness in connexin 40-deficient mice. Am. J. Physiol. Ren. Physiol. 304, F1181–F1186. 10.1152/ajprenal.00721.2012 PMC3651628 23445620

[B28] Pogoda K. Kameritsch P. Mannell H. Pohl U. (2019). Connexins in the control of vasomotor function. Acta Physiol. (Oxf). 225, e13108. 10.1111/apha.13108 29858558

[B29] Pohl U. (2020). Connexins: key players in the control of vascular plasticity and function. Physiol. Rev. 100, 525–572. 10.1152/physrev.00010.2019 31939708

[B30] Rauch C. Feifel E. Kern G. Murphy C. Meier F. Parson W. (2018). Differentiation of human iPSCs into functional podocytes. PloS one 13, e0203869. 10.1371/journal.pone.0203869 30222766 PMC6141081

[B31] Rignault S. Haefliger J. A. Gasser D. Markert M. Nicod P. Liaudet L. (2005). Sepsis up-regulates the expression of connexin 40 in rat aortic endothelium. Crit. Care Med. 33, 1302–1310. 10.1097/01.ccm.0000165968.47343.0d 15942348

[B32] Saez J. C. Berthoud V. M. Branes M. C. Martinez A. D. Beyer E. C. (2003). Plasma membrane channels formed by connexins: their regulation and functions. Physiol. Rev. 83, 1359–1400. 10.1152/physrev.00007.2003 14506308

[B33] Sandow S. L. Looft-Wilson R. Doran B. Grayson T. H. Segal S. S. Hill C. E. (2003). Expression of homocellular and heterocellular gap junctions in hamster arterioles and feed arteries. Cardiovasc Res. 60, 643–653. 10.1016/j.cardiores.2003.09.017 14659810

[B34] Siddiqui M. Swarbreck S. Shao Q. Secor D. Peng T. Laird D. W. (2015). Critical role of Cx40 in reduced endothelial electrical coupling by lipopolysaccharide and hypoxia-reoxygenation. J. Vasc. Res. 52, 396–403. 10.1159/000445772 27194161

[B35] Sorensen C. M. Cupples W. A. (2019). Myoendothelial communication in the renal vasculature and the impact of drugs used clinically to treat hypertension. Curr. Opin. Pharmacol. 45, 49–56. 10.1016/j.coph.2019.04.005 31071677

[B36] Takenaka T. Inoue T. Kanno Y. Okada H. Meaney K. R. Hill C. E. (2008). Expression and role of connexins in the rat renal vasculature. Kidney Int. 73, 415–422. 10.1038/sj.ki.5002673 18046320

[B37] Tyml K. (2011). Role of connexins in microvascular dysfunction during inflammation. Can. J. Physiol. Pharmacol. 89, 1–12. 10.1139/y10-099 21186372

[B38] van Rijen H. V. van Kempen M. J. Postma S. Jongsma H. J. (1998). Tumour necrosis factor alpha alters the expression of connexin43, connexin40, and connexin37 in human umbilical vein endothelial cells. Cytokine 10, 258–264. 10.1006/cyto.1997.0287 9617570

[B39] Wang W. Tang Y. Ni L. Kim E. Jongwutiwes T. Hourvitz A. (2012). Overexpression of Uromodulin-like1 accelerates follicle depletion and subsequent ovarian degeneration. Cell death Dis. 3, e433. 10.1038/cddis.2012.169 23190605 PMC3542605

[B40] Zechariah A. Tran C. H. T. Hald B. O. Sandow S. L. Sancho M. Kim M. S. M. (2020). Intercellular conduction optimizes arterial Network function and conserves blood flow homeostasis during cerebrovascular challenges. Arterioscler. Thromb. Vasc. Biol. 40, 733–750. 10.1161/ATVBAHA.119.313391 31826653 PMC7058668

[B41] Zehra T. Cupples W. A. Braam B. (2021). Tubuloglomerular feedback synchronization in nephrovascular Networks. J. Am. Soc. Nephrol. 32, 1293–1304. 10.1681/ASN.2020040423 33833078 PMC8259654

